# Could ALDH2^*^2 be the reason for low incidence and mortality of ovarian cancer for East Asia women?

**DOI:** 10.18632/oncotarget.23605

**Published:** 2017-12-22

**Authors:** Shaomin Yan, Guang Wu

**Affiliations:** ^1^ Bioscience and Technology Research Center, Guangxi Academy of Sciences, Nanning, Guangxi, 530007, China

**Keywords:** aldehyde dehydrogenase 2, ALDH2^*^2, East Asian, stem cell, ovarian cancer

## Abstract

It is curious that East Asian women have a low incidence and mortality of ovarian cancer in various epidemiological studies. Although different explanations were given, they appear unsubstantial. We notice that East Asian population usually are inactive aldehyde dehydrogenase 2 mutation (ALDH2^*^2) carriers, and ALDH plays an important role in the resistance of ovarian cancer to chemotherapeutics, especially in ovarian cancer stem cells. Therefore, we hypothesize whether ALDH2 mutation is the major reason for low incidence and mortality of ovarian cancer in East Asian women, and use the evidence from literature, transcriptomic data with average 5-year overall survival to confirm our hypothesis.

## INTRODUCTION

Ovarian cancer is the seventh most common cancer in women and is the eighth most frequent cause of cancer death among women [1]. The occurrence of ovarian cancer has been related to many factors, for example, age of menarche [2, 3], short or irregular cycles [2–6], age of menopause [7, 8], age of the first birth [2, 3, 9, 10], age of last pregnancy [7, 10, 11], the number of children [2, 3, 7, 10, 11], period of breastfeeding [12–16], oral contraceptives [17–21], intake of phytochemicals [22], genes BRCA1 or BRCA2 [23], etc.

As many diseases show clear patterns in their geographic and race distributions, ovarian cancer also has preference in these regards, for example, the women from East Asia have a very low incidence rate and the lowest mortality rate [1, 24]. Death due to ovarian cancer is more common in North America and Europe than in Africa and Asia [1]. High rates of epithelial ovarian cancer are reported in industrialized nations with the exception of Japan [24], which may be due to diet in those countries.

### Ovarian cancer and its stem cell

As ovarian cancer is a high death-to-incidence disease, the role of cancer stem cells in ovarian cancer cannot be ignored because cancer stem cells are frequently resistant to chemotherapeutic and radiation treatments [25]. For example, drug resistance is due to the fact that specific therapies enrich cancer stem cells in residual pancreatic cancer treated with gemcitabine [26], colorectal cancer treated with cyclophosphamide [27], hepatocellular carcinoma treated with doxorubicin and fluorouracil [28] and lung cancer treated with cisplatin, doxorubicin, and methotrexate [29].

Ovarian cancer has a great degree of heterogeneity because it may arise from germ cell, stromal, or epithelial compartments [24]. Similarly, ovarian cancer is the best example of intra-tumor heterogeneity of cancer stem cell [30, 31]. It was hypothesized that ovarian cancer is driven and sustained by cancer stem cell as shown by CD44+/CD24- [32, 33], CD117 and CD133 [34, 35], especially ALDH1A1 [36–38]. Indeed, ALDH+/CD133+ ovarian cancer primary cells were defined as the top of hierarchical structure in ovarian cancer and as stem cell markers. This combination is a more multipotent phenotype than others including ALDH-/CD133+ [39]. Furthermore, it was reported that ALDH1 is better than CD133 in terms of identification of primary ovarian carcinoma-derived cells, which express stemness genes and are capable of self-renewal and tumor initiation [40].

As a marker of stem cells in both normal tissues and cancers [41], ALDH1 plays an extremely important role in ovarian cancer stem cells [25, 42] because studies show that ALDH1 activity is directly related to a subpopulation of ovarian cancer cells with cancer stem cell-like properties [36, 37, 39, 43–45]. Of ALDH1 isozymes (ALDH1A1, ALDH1A2 and ALDH1A3), expression of ALDH1A1 is prominent in cancer stem cell [42], for instance, in human breast cancer cell lines [46]. The role of ALDH1A1 in ovarian cancer stem cells, although it is not fully clear, is not far from its nominal role, i.e. its detoxifying role in terms of preventing the accumulation of reactive oxygen species and of reactive aldehydes. Additional role of ALDH1A1 is its regulatory function on ATP-binding cassette (ABC) drug transporters, which in turn leads to the resistance of ovarian cancer to chemotherapeutics [47].

It was found that ovarian cancer patients, who have high levels of type I receptor tyrosine kinase-like orphan receptor (ROR1), have stem cell-like gene-expression signatures, and their relapse rate and median survival are higher and shorter than other patients. The importance is that ROR1-positive (ROR1+) cells also expressed ALDH1 [48]. In ovarian cancer stem cells, ALDH1 have relatively high enzymatic activity [38, 49, 50], which no doubt intensifies detoxification of intracellular aldehydes as well as cytotoxic drugs [41, 51]. Accordingly, ALDH1 offers resistance to chemotherapeutics and radiation therapy [45]. Such role was confirmed by the finding that the knockdown of ALDH1A1 gene in ovarian cancer cell lines can restore the ovarian cancer cells’ sensitivity to chemotherapy *in vitro* [47] and xenograft models in mouse [38]. Therefore, the role of ALDH1 in ovarian cancer stem cells is far beyond the detoxification.

Regulation of ALDH1A1 in ovarian cancer stem cells is suggested at the transcriptional level through Wnt/β-catenin pathway [52]. Knockdown of ALDH1A1 in ovarian cancer cell line A2780 decreases regulators KLF4 and p21, which are the cell cycle checkpoints, and leads to an enhanced cell proliferation. This is the anti-proliferative role of ALDH1A1 because actively proliferating cells are more subject to cytotoxic drugs and so loss of ALDH1A1 contributes to the sensitization of ovarian carcinoma cells to chemotherapy [53] although a study showed the difference due to interplay between ALDH1A1 and the stemness-associated gene SOX2 [40]. Also, loss of ALDH1A1 triggered DNA damage suggesting that ALDH1A1 plays a genome-protecting role in ovarian cancer stem cells [53]. As a result, it is suggested that ALDH1A1 could be potential therapeutic target because a small-molecule ALDH1A1 inhibitor abolished sphere formation in ovarian cancer [52].

## ALDH2

Apart from research in ALDH1 (ALDH1A1) in cancer stem cells, in fact, research interests in ALDH have been greatly increased recently [54], especially, ALDH2 [55], because ALDH2 is the most efficient enzyme for the metabolism of ethanol-derived acetaldehyde with the lowest *K*_*m*_ [56]. ALDH has three different classes in mammals: class 1 (low *K*_*m*_, cytosolic), class 2 (low *K*_*m*_, mitochondrial), and class 3 (high *K*_*m*_, such as those expressed in tumors, stomach, and cornea). In all three classes, constitutive and inducible forms exist. ALDH1 and ALDH2 are the most important enzymes for aldehyde oxidation, and both are tetrameric enzymes composed of 54 kDa subunits.

The enzymatic reaction catalyzed by ALDH seems to be simple in humans, i.e. alcohol dehydrogenase (ADH) catalyzes ingested ethanol to acetaldehyde, and ALDH, mainly mitochondrial enzyme ALDH2, catalyzes acetaldehyde into acetate. Therefore, a single point mutation in ALDH2, termed ALDH2^*^2, wherein a lysine residue replaces a glutamate in the active site at position 487 of ALDH2 [57], causes facial flushing, headaches, nausea, dizziness, and cardiac palpitations in humans after alcohol consumption [58–61] because aldehydes cannot be fully detoxified [62]. Homozygous individuals with the mutant allele have almost no ALDH2 activity, and those heterozygous for the mutation have reduced activity. Thus, the mutation is partially dominant.

What is less known is that ALDH2 metabolizes numerous short-chain aliphatic aldehydes, aromatic and polycyclic aldehydes [63], environmental aldehydes such as acrolein in tobacco smoke and in car exhaust, and particularly endogenous aldehydic products from lipid peroxidation under oxidative stress, such as 4-hydroxy-2-nonenal (4-HNE) and malondialdehyde (MDA) [64, 65].

Consequently, many diseases and health problems can be related to ALDH2 mutation, whose carriers were associated to myocardial infarction [66], impaired myocardial function in rodents and humans [65, 67, 68], hypertension [69], blood pressure variation in East Asians [70], non-insulin-dependent diabetes mellitus due to the maternal ALDH2 [71, 72], a higher incidence of Alzheimer’s disease in Asian patients with the inactivating ALDH2^*^2 mutation [73–75], increase of esophageal cancer risk no matter of alcoholic beverage drinkers or not [76].

Intriguingly, the ALDH2^*^2 mutation exists mainly in 560 million East Asians [61, 77, 78] rather than the rest parts of the world [79]. This is because most Caucasians have both active ALDH1 and ADLH2, while approximately 50% of East Asians have active ALDH1 but not active ALDH2 due to its mutation. Therefore, flushing symptom is more popular for East Asians than for Caucasians, and the increased exposure to acetaldehyde in individual may be more susceptible to many types of cancer [55]. Many other studies from Japan, Taiwan, and China have overwhelmingly confirmed the significant association between ALDH2 enzyme deficiency and upper aerodigestive track (oropharyngolaryngeal, esophageal, stomach, colon and lung) cancer risk [80–86].

In broader sense, high ALDH expression is associated with a poor prognosis in acute myeloid leukaemia [87, 88], breast cancer [46, 89–91], early-stage lung cancer [92], head and neck squamous cell carcinoma [93], pancreas cancer [94], and prostate cancer [95]. Although ALDH2^*^2 mutation is associated with various diseases [55], there are exceptions, for example, liver cancer [96], Parkinson’s disease [97], and stroke [98, 99].

To go further, one can find the difference between male and female ALDH2^*^2 carriers with respect to different diseases. Chinese male ALDH2^*^2 carriers have a significantly higher incidence of acute coronary syndrome than noncarriers [100]. Also it demonstrated that the ALDH2^*^2 genotype is a risk factor for myocardial infarction in Japanese men [101]. On the other hand, ALDH2^*^2 genotype seemed to be a risk factor for non-insulin-dependent diabetes mellitus in women, but not in men [102]. Premenopausal females have a lower risk for cardiovascular disease, because female hearts have increased phosphorylation and activity of ALDH2 in ischemia and reperfusion injury, so ALDH2 activator is more effective in males than in females, and inhibition blocks the phosphorylation of ALDH2 in females, but had no effect in males [103]. A study including 2,200-plus Japanese between 40 and 70 years showed a clear higher level of serum lipid peroxides in female ALDH2^*^2 carriers after exclusion of alcohol drinking behavior [104].

If this ALDH2^*^2 mutation is considered so harmful [55], why it has been so popular in East Asia existing for 2000–3000 years [105]. What is the advantage in evolution to keep this mutation although unanswered hypotheses have been given [106, 107] ? In the past, ALDH2 mutation was considered as benign because it is a limiting factor for over drinking of alcoholic beverage [61, 108–110]. Another piece of evidence that shows the positive aspect of ALDH2 with clear difference between males and females is the ALDH2^*^2 genotype was associated with a longer life for male Koreans [111]. In addition, there is difference in ALDS2^*^2 carriers with certain disease in East Asia with respect to locality, for example, the number of Fanconi anemia patients with ALDH2^*^2 in Japan and Korea [112, 113] is far higher than in China or Taiwan, where the percentage of ALDH2^*^2 carriers is higher i.e., > 40% in Taiwan.

Over here, comparison between East Asian women with East Asian men is to indicate that ALDH2 plays different roles in East Asian women and men with respect to different diseases, so we could not simply say ALDH2 bad or good. On the other hand, one may wonder why we do not refer studies on East Asian women with Caucasian women. Actually, our theme begins to mention that East Asian women have a low incidence and mortality of ovarian cancers, which is the comparison between East Asian women with Caucasian women as well as women from the rest parts of world. Although a huge amount studies have been done to compare East Asian women with Caucasian women with respect to various diseases, there is no study conducted on ovarian cancer with respect to ALDH2. This is why we cannot conduct a meta-analysis to combine such studies from all the centers, whereas we can only propose a deduced hypothesis. Furthermore, one may also wonder who will conduct such a study to compare East Asian women with Caucasian women on ALDH2, simply because East Asian women generally do not have ALDH2.

Very interestingly and curiously, the relationship between ALDH2 and ovarian cancer has drawn little attention. Indeed, in transgenic mouse, the ALDH2^*^2 mutant subunits overexpressed particularly notably in cardiac and skeletal muscles [114] rather than ovary. Alcohol consumption does not appear to be related to ovarian cancer [115].

Taken all the references together, it is highly likely that the very low incidence rate and the lowest mortality rate of ovarian cancer in East Asian women is mainly due to the fact that many East Asian women are ALDH2^*^2 carriers. Therefore the ovarian stem cancer cells in East Asian women are more susceptible to environmental and endogenous aldehydic products at early stage of ovarian cancer and to chemotherapy at later stage of ovarian cancer, and these “toxic” substances could kill cancer stem cells readily. These are reasonable because ALDH cannot actively detoxify these substances in East Asian women who carry ALDH2^*^2. Indeed, although 4-HNE is capable to covalently bind to DNA as an important factor of carcinogenesis, it is also cytotoxic for cancer cells and can modulate their growth [116]. Thus ALDH1A1 inhibitor [41, 52] and eradication of ALDH high expression cells [117–121] were advocated although ALDH2 was not mention. From laboratory viewpoint, the determination of ALDH1A1 activity in live cells and of isolating ALDH1-positive cells with a fluorescence-based assay [122] is perhaps easier than determination of ALDH2, which is located in mitochondria although it is more active than ALDH1. Very strictly speaking, the study should be conducted in such a way that includes only East Asian women with ALDH2^*^2 versus ALDH2 with respect to their incidence and mortality of ovarian cancer. Therefore, we do not have direct experimental evidence to connect ALDH2 with ovarian cancer, but we can only deduce such a relationship using various pieces of knowledge in literature, which is the reason of why we propose our hypothesis and hope such hypothesis can stimulate more discussions and experiments. Fortunately, the transcriptomic data can provide experimental evidence for ALDH2 activity in cancer patients, which provide additional support for our literature evidence in the next section.

Another piece of evidence to support ALDH2’s role in ovarian cancer is that the risk of ovarian cancer goes up with age, and ALDH2^*^2 homozygous genotype was significantly reduced in females in the 60–70s age group versus 40–50s group in a study with more than 2,200 Japanese [104].

One may argue that our guess based on several paragraphs, however Newton’s guess on gravity was only based on the fact that an apple falls from tree. Therefore, the importance of guess is not dependent on how many paragraphs but on deduction. Moreover our guess is supported by fully referenced literature, which shows the ALDH’s role in cancer stem cells, as a component of ALDH, ALDH2 should play the same role as ALDH does although it was not on clinical routine screening now.

### Evidence from cancer stem cells

Ideally the best way to test our reasoning and rationale is to compare the ALDH2 activity in ovarian cancer stem cells with the killing rate of chemotherapy for ovarian cancer stem cells or compare the ALDH2 activity in ovarian stem cells with the ovarian cancer incidence. Currently, this could be impossible. To take a step back, we analyze all the available transcriptomic data (GSE19713 [123], GSE23806 [124–126], GSE28799 [127], GSE35603 [128], GSE67966) of different cancer stem cells in Gene Expression Omnibus (GEO) [129, 130] using platform GPL570 [131] with ALDH1A1 and ALDH2 expression against 5-year overall survival data in different cancers documented in literature (Supplementary Table 1), if we consider ALDH1A1 and ALDH2 expression somewhat similar to their activities.

**Figure 1:**
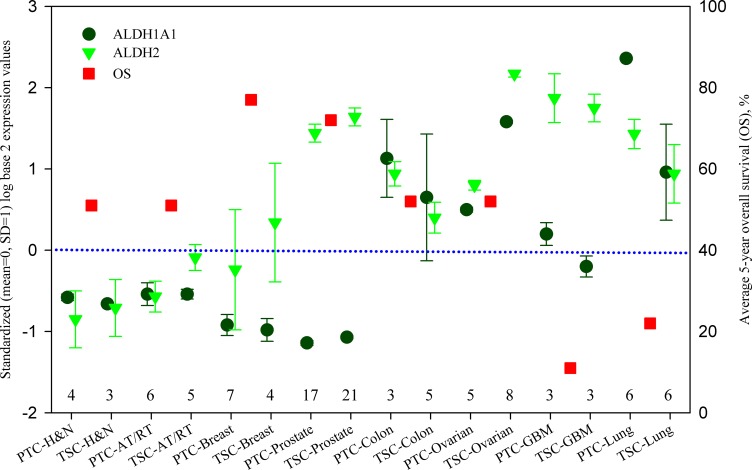
ALDH1A1 and ALDH2 expression of parental tumor cells and tumor stem-like cells, and 5-year overall survival in different cancers ALDH1A1 and ALDH2 are presented by standardized (mean = 0, SD = 1) log base 2 expression values at the left axis. The data were calculated from the Series GSE19713, GSE23806, GSE28799, GSE35603 and GSE67966, and their sample numbers are listed above the x-axis. Average 5-year overall survival (OS) data are obtained from the literatures listed in Supplementary Table 1 and presented at the right axis. PTC: parental tumor cell, TSC: tumor stem-like cell, AT/RT: Atypical teratoid/rhabdoid tumour, GBM: glioblastoma, H & N: head and neck.

Figure 1 shows this analysis. In this figure, samples of eight types of cancers are further divided into parental tumor cell (PTC) and tumor stem-like cell (TSC), so there are 16 labels along *x*-axis. ALDH1A1 and ALDH2 expressions are presented as circles and triangles. As can be seen, the expression of ALDH1A1 and ALDH2 vary in different cancers, as well as between ovarian cancer PTC and TSC. ALDH expressions, especially the expression of ALDH1A1, are low in atypical teratoid/rhabdoid tumour and cancers from head and neck, breast, and prostate, but high in the rest of cancers. Moreover, the average 5-year overall survival (OS) is presented in this figure as square symbol for 8 different cancers because OS does not distinguish PTC from TSC.

A general trend can be found in Figure 1, that is, OS is higher when the expressions of ALDH1A1 and ALDH2 are lower, whereas OS is lower when the expressions of ALDH1A1 and ALDH2 are higher. This feature is consistent with previous studies [46, 87, 88, 89–95] and can suggest the detoxification of ALDH on chemotherapy. Although prostate cancer has high ALDH2 expression with high OS, its ALDH1A1 expression is indeed very low. Because ALDH1A1 has an anti-proliferative role, a low activity of ALDH1A1 can promote cell proliferation and increase the sensitivity of cancer cells to chemotherapy [53]. Yet, great caution should be paid to OS because we could not stratify OS according to the treatments of chemotherapy, radiotherapy and surgery. Also the baseline of OS is unknown so OS in ovarian cancer apparently is relatively high.

Comparing the expression between ALDH1A1 and ALDH2, stronger ALDH2 expression can be found in the tumor stem-like cells of atypical teratoid/rhabdoid tumour (TSC-AT/RT), and in both parental and stem-like tumor cells of breast cancer, prostate cancer, ovarian cancer and glioblastoma (GBM). This observation is in good agreement with the knowledge that ALDH2 plays a more important role on detoxification [56]. It is notable to see that ALDH2 expression is at very high level in stem-like tumor cells of ovarian cancer, indicating that a high ALDH2 activity of stem cells renders significant influence on the morbidity and mortality of ovarian cancer. Therefore the population who lack ALDH2 would be more sensitive to chemotherapy and internal toxic substances such as 4-HNE. Along this thought of line, we would have expected to see a high OS in East Asian women with ovarian cancer because their ALDH2 activity would be zero in ALDH2 mutation carriers. Therefore, our hypothesis is supported by all the available transcriptomic data on ovarian cancer stem cells.

### Final remark

In this review, we use the evidence from literature, transcriptomic data with average 5-year overall survival to suggest that the key factor that determines the low incidence and mortality of ovarian cancer in East Asian women is the ALDH2 mutation.

Interestingly, East Asia with its ALDH2^*^2 mutation seems to be the most economically active place in the world for quite a considerable periods in human history. This leads to the question of whether ALDH2^*^2 mutation is helpful to intelligent development.

## SUPPLEMENTARY MATERIALS TABLE





## Abbreviations

4-HNE: 4-hydroxy-2-nonenal
ABC: ATP-binding cassette
ADH: alcohol dehydrogenase
ALDH: aldehyde dehydrogenase
GEO: Gene Expression Omnibus
MDA: malondialdehyde
OS: average 5-year overall survival,
PTC: parental tumor cell
ROR1: type I receptor tyrosine kinase-like orphan receptor
TSC: tumor stem-like cell
