# Supplementary material for: Could ALDH2*2 be the reason for low incidence and mortality of ovarian cancer for East Asia women?
Source: Oncotarget. 2017 Dec 22;9(15):12503–12. doi: 10.18632/oncotarget.23605 (PMC5844765; doi:10.18632/oncotarget.23605)
Supplement: Supplementary file 1 [file oncotarget-09-12503-s001.pdf]

Could ALDH2*2 be the reason for low incidence and mortality of ovarian cancer for East Asia women?

SUPPLEMENTARY MATERIALS

Supplementary Table 1: Average 5-year overall survival in different cancers

Cancer	Average 5-year overall survival (%)	Reference (PMID)
Atypical teratoid/rhabdoid tumour	51 (28–89)	[1–5]
Breast cancer	77 (14–100)	[6–13]
Colon cancer	52 (5–80)	[12, 14–28]
Glioblastoma	11 (3–31)	[29–40]
Lung cancer	22 (5–70)	[12, 41–51]
Oral cancer	51 (21–75)	[52–64]
Ovarian cancer	52 (37–85)	[65–74]
Prostate cancer	72 (14–100)	[12, 75–82]
