# Supplementary material for: Could ALDH2*2 be the reason for low incidence and mortality of ovarian cancer for East Asia women?
Source: Oncotarget. 2017 Dec 22;9(15):12503–12. doi: 10.18632/oncotarget.23605 (PMC5844765; doi:10.18632/oncotarget.23605)
Supplement: Supplementary file 2 [file oncotarget-09-12503-s002.pdf]

Could ALDH2*2 be the reason for low incidence and mortality of ovarian cancer for East Asia women?

SUPPLEMENTARY MATERIALS

REFERENCES

1. Odagiri K, Omura M, Hata M, Aida N, Niwa T, Goto H, Ito S, Adachi M, Yoshida H, Yuki H, Inoue T. Treatment outcomes and late toxicities in patients with embryonal central nervous system tumors. Radiat Oncol. 2014; 9:201.
2. Ostrom QT, Chen Y, M de Blank P, Ondracek A, Farah P, Gittleman H, Wolinsky Y, Kruchko C, Cohen ML, Brat DJ, Barnholtz-Sloan JS. The descriptive epidemiology of atypical teratoid/rhabdoid tumors in the United States, 2001-2010. Neuro Oncol. 2014; 16:1392–9.
3. Moschovi M, Koultouki E, Stefanaki K, Sfakianos G, Tourkantoni N, Prodromou N, Alexiou GA. Prognostic significance of angiogenesis in relation to Ki-67, p-53, p-27, and bcl-2 expression in embryonal tumors. Pediatr Neurosurg. 2011; 47:241–7.
4. Woehrer A, Slavc I, Waldhoer T, Heinzl H, Zielonke N, Czech T, Benesch M, Hainfellner JA, Haberler C; Austrian Brain Tumor Registry. Incidence of atypical teratoid/rhabdoid tumors in children: a population-based study by the Austrian Brain Tumor Registry, 1996-2006. Cancer. 2010; 116:5725–32.
5. Meyers SP, Khademian ZP, Biegel JA, Chuang SH, Korones DN, Zimmerman RA. Primary intracranial atypical teratoid/rhabdoid tumors of infancy and childhood: MRI features and patient outcomes. AJNR Am J Neuroradiol. 2006; 27:962–71.
6. Kim SY, Han BK, Kim EK, Choi WJ, Choi Y, Kim HH, Moon WK. Breast Cancer Detected at Screening US: Survival Rates and Clinical-Pathologic and Imaging Factors Associated with Recurrence. Radiology. 2017; 6:162348.
7. McCrady JD, Pendery GB, Camp BJ, Clark DR, Smith BP, Davis RH Jr, Moody GM. The effects of hemolyzed blood on pulmonary and systemic arterial pressure and heart rate of the dog. Can J Comp Med. 1978; 42:69–73.
8. Kruszewski FH, Hennings H, Yuspa SH, Tucker RW. Regulation of intracellular free calcium in normal murine keratinocytes. Am J Physiol. 1991; 261:C767–73.
9. McKee M. Health statistics on the Internet. J R Soc Med. 1998; 91:399.
10. Weale RA. High light intensities and photo-chemical reactions of human visual pigments in situ. Cold Spring Harb Symp Quant Biol. 1965; 30:335–43.
11. Lohrisch C, Jackson J, Jones A, Mates D, Olivotto IA. Relationship between tumor location and relapse in 6,781 women with early invasive breast cancer. J Clin Oncol. 2000; 18:2828–35.
12. Ottawa (ON): Canadian Agency for Drugs and Technologies in Health; 2015 Sep. CADTH Rapid Response Reports. Low-Dose Computed Tomography for Lung Cancer Screening: A Review of the Clinical Effectiveness, Diagnostic Accuracy, Cost-Effectiveness, and Guidelines [Internet].
13. Kulik U, Lehner F, Bektas H, Klempnauer J. Liver Resection for Non-Colorectal Liver Metastases - Standards and Extended Indications. Viszeralmedizin. 2015; 31:394–8.
14. Collins D, Chua H. Contemporary surgical management of synchronous colorectal liver metastases. F1000Res. 2017; 6:598.
15. Haram A, Boland MR, Kelly ME, Bolger JC, Waldron RM, Kerin MJ. The prognostic value of neutrophil-to-lymphocyte ratio in colorectal cancer: A systematic review. J Surg Oncol. 2017; 115:470–479.
16. Zhao JM, Wang YH, Yao N, Wei KK, Jiang L, Hanif S, Wang ZX. Poor Prognosis Significance of Pretreatment Thrombocytosis in Patients with Colorectal Cancer: a Meta-Analysis. Asian Pac J Cancer Prev. 2016; 17:4295–4300.
17. Schernberg A, Servagi-Vernat S, Loganadane G3, Touboul E3, Bosset JF, Huguet F3. Rectal squamous cell carcinoma treatment: Retrospective experience in two French university hospitals, review and proposals. Cancer Radiother. 2016; 20:824–829.
18. Mozdiak E, Tsertsvadze A3, McFarlane M, Widlak M, Tabuso M, Dunlop A, Arasaradnam R,5. The effect of the 2-week wait referral system on the detection of and mortality from colorectal cancer: protocol of a systematic review and meta-analysis. Syst Rev. 2016; 5:182.
19. Sabanathan D, Eslick GD, Shannon J. Use of Neoadjuvant Chemotherapy Plus Molecular Targeted Therapy in Colorectal Liver Metastases: A Systematic Review and Meta-analysis. Clin Colorectal Cancer. 2016; 15:e141-e147.
20. Kim JS, Kim YG, Park EJ, Kim B, Lee HK, Hong JT, Kim Y, Han SB. Cell-based Immunotherapy for Colorectal Cancer with Cytokine-induced Killer Cells. Immune Netw. 2016; 16:99–108.
21. Pagès PB, Le Pimpec-Barthes F, Bernard A3. Surgery for pulmonary metastases from colorectal cancer: Predictive factors for survival. Rev Mal Respir. 2016; 33:838–852.

22. Mondaca S, Villalón C, Leal JL, Zúñiga Á, Bellolio F, Padilla O, Palma S, Garrido M, Nervi B. Benefit of adjuvant 5-fluorouracil based chemotherapy for colon cancer: a retrospective cohort study. Rev Med Chil. 2016; 144:145–51.
23. Liu W, Zhou JG, Sun Y, Zhang L, Xing BC. The role of neoadjuvant chemotherapy for resectable colorectal liver metastases: a systematic review and meta-analysis. Oncotarget. 2016; 7:37277–37287. https://doi.org/10.18632/oncotarget.8671.
24. Ong ML, Schofield JB. Assessment of lymph node involvement in colorectal cancer. World J Gastrointest Surg. 2016; 8:179–92.
25. Hadden WJ, de Reuver PR, Brown K3, Mittal A3, Samra JS3, Hugh TJ4. Resection of colorectal liver metastases and extra-hepatic disease: a systematic review and proportional meta-analysis of survival outcomes. HPB (Oxford). 2016; 18:209–20.
26. Zhang X, Shao S, Gao Y, Zhang M, Lu Y3. Meta-analysis of relationship between extranodal tumor deposits and prognosis in patients with colorectal cancer. Zhonghua Wei Chang Wai Ke Za Zhi. 2016; 19:334–8.
27. Ehrlich A, Kairaluoma M, Böhm J, Vasala K3, Kautiainen H, Kellokumpu I5. Laparoscopic Wide Mesocolic Excision and Central Vascular Ligation for Carcinoma of the Colon. Scand J Surg. 2016; pii: 1457496915613646.
28. Brown KM, Geller DA,3. What is the Learning Curve for Laparoscopic Major Hepatectomy? J Gastrointest Surg. 2016; 20:1065–71.
29. Kulik U, Lehner F, Bektas H, Klempnauer J. Liver Resection for Non-Colorectal Liver Metastases - Standards and Extended Indications. Viszeralmedizin. 2015 Dec; 31:394–8.
30. Batash R, Asna N, Schaffer P3, Francis N, Schaffer M2. Glioblastoma Multiforme, Diagnosis and Treatment; Recent Literature Review. Curr Med Chem. 2017 May 16.
31. Ohba S, Hirose Y. Current and Future Drug Treatments for Glioblastomas. Curr Med Chem. 2016; 23:4309–4316.
32. Espinoza JC, Haley K, Patel N, Dhall G, Gardner S, Allen J, Torkildson J, Cornelius A, Rassekh R, Bedros A, Etzl M, Garvin J, Pradhan K, et al. Outcome of young children with high-grade glioma treated with irradiation-avoiding intensive chemotherapy regimens: Final report of the Head Start II and III trials. Pediatr Blood Cancer. 2016; 63:1806–13.
33. Delgado-López PD, Corrales-García EM. Survival in glioblastoma: a review on the impact of treatment modalities. Clin Transl Oncol. 2016; 18:1062–1071.
34. Eder K, Kalman B. The Dynamics of Interactions Among Immune and Glioblastoma Cells. Neuromolecular Med. 2015; 17:335–52.
35. Rehman AA, Elmore KB, Mattei TA. The effects of alternating electric fields in glioblastoma: current evidence on therapeutic mechanisms and clinical outcomes. Neurosurg Focus. 2015; 38:E14.
36. Patel MA, Kim JE, Ruzevick J, Li G, Lim M. The future of glioblastoma therapy: synergism of standard of care and immunotherapy. Cancers (Basel). 2014; 6:1953–85.
37. Ostrom QT, Bauchet L, Davis FG, Deltour I, Fisher JL, Langer CE, Pekmezci M, Schwartzbaum JA, Turner MC, Walsh KM, Wrensch MR, Barnholtz-Sloan JS. The epidemiology of glioma in adults: a "state of the science" review. Neuro Oncol. 2014; 16:896–913.
38. Field KM, Rosenthal MA, Yilmaz M, Tacey M, Drummond K. Comparison between poor and long-term survivors with glioblastoma: review of an Australian dataset. Asia Pac J Clin Oncol. 2014; 10:153–61.
39. Badiyan SN, Markovina S, Simpson JR, Robinson CG, DeWees T, Tran DD, Linette G, Jalalizadeh R, Dacey R, Rich KM, Chicoine MR, Dowling JL, Leuthardt EC, et al. Radiation therapy dose escalation for glioblastoma multiforme in the era of temozolomide. Int J Radiat Oncol Biol Phys. 2014; 90:877–85.
40. Li M, Deng H, Peng H, Wang Q. Functional nanoparticles in targeting glioma diagnosis and therapies. J Nanosci Nanotechnol. 2014; 14:415–32.
41. Saito S, Espinoza-Mercado F, Liu H, Sata N, Cui X, Soukiasian HJ2. Current status of research and treatment for non-small cell lung cancer in never-smoking females. Cancer Biol Ther. 2017; 11:1–10.
42. Schvartsman G, Ferrarotto R, Massarelli E3. Checkpoint inhibitors in lung cancer: latest developments and clinical potential. Ther Adv Med Oncol. 2016; 8:460–473.
43. Hata A, Suzuki H, Nakajima T, Tanaka K, Fujiwara T, Wada H, Iwata T, Yoshida S, Yoshino I. Concomitant Interstitial Lung Disease Is a Risk Factor for Pleural Invasion in Lung Cancer. Ann Thorac Surg. 2017; 103:967–974.
44. Gao XL, Zhang KW, Tang MB, Zhang KJ, Fang LN, Liu W2. Pooled analysis for surgical treatment for isolated adrenal metastasis and non-small cell lung cancer. Interact Cardiovasc Thorac Surg. 2017; 24:1–7.
45. Okumura M. Trends and current status of general thoracic surgery in Japan revealed by review of nationwide databases. J Thorac Dis. 2016; 8:S589–95.
46. Hirsch FR, Scagliotti GV, Mulshine JL, Kwon R, Curran WJ Jr, Wu YL, Paz-Ares L7. Lung cancer: current therapies and new targeted treatments. Lancet. 2017 Jan 21; 389:299–311.
47. Swedish Council on Health Technology Assessment. Computed Tomography in Screening for Lung Cancer [Internet].
48. Mizuno K, Mataki H, Seki N, Kumamoto T, Kamikawaji K, Inoue H. MicroRNAs in non-small cell lung cancer and idiopathic pulmonary fibrosis. J Hum Genet. 2017; 62:57–65.
49. Shi JG, Shao HJ, Jiang FE, Huang YD. Role of radiation therapy in lung cancer management - a review. Eur Rev Med Pharmacol Sci. 2016; 20:3217–22.
50. De Ruysscher D, Lueza B, Le Péchoux C, Johnson DH, O'Brien M, Murray N, Spiro S, Wang X, Takada M, Lebeau B, Blackstock W1, Skarlos D1, Baas P, et al. RTT-SCLC Collaborative Group. Impact of thoracic radiotherapy timing in limited-stage small-cell lung cancer: usefulness of

the individual patient data meta-analysis. Ann Oncol. 2016; 27:1818–28.

51. Prokop M. Lung cancer screening: the radiologist's perspective. Semin Respir Crit Care Med. 2014; 35:91–8.
52. Liao CT, Lee LY, Hsueh C, Lin CY, Fan KH, Wang HM, Hsieh CH, Ng SH, Lin CH, Tsao CK, Kang CJ, Fang TJ, Huang SF, et al. Clinical Outcomes in pT4 Tongue Carcinoma are Worse than in pT3 Disease: How Extrinsic Muscle Invasion Should be Considered? Ann Surg Oncol. 2017 Jun 3.
53. Chow TL, Kwan WWY, Fung SC, Ho LI2. Prognostic value of lymph node density in buccal squamous cell carcinoma. Am J Otolaryngol. 2017; pii:S0196-070930620-2.
54. Ikawa H, Koto M, Takagi R, Ebner DK, Hasegawa A, Naganawa K, Takenouchi T, Nagao T, Nomura T, Shibahara T, Tsuji H, Kamada T2. Prognostic factors of adenoid cystic carcinoma of the head and neck in carbon-ion radiotherapy: The impact of histological subtypes. Radiother Oncol. 2017; 123:387–393.
55. Pan H, Li T, Huang Z, Yu H, Kong D, Ding Y, Pan C, Jiang Y4. Laparoscopic versus opengastric surgery for the treatment of pathological T1N0M0 gastric cancer in elderly patients: a matched study. Sci Rep. 2017; 7:1919.
56. Nakajima Y, Iijima Y, Kinoshita H, Akiyama H, Beppu T, Uramoto H, Hirata T. Surgical Treatment for Pulmonary Metastasis of Head and Neck Cancer: Study of 58 Cases. Ann Thorac Cardiovasc Surg. 2017.
57. Sommers LW, Steenbakkers RJHM, Bijl HP, Vemer-van den Hoek JGM, Roodenburg JLN, Oosting SF, Halmos GB, de Rooij SE, Langendijk JA2. Survival Patterns in Elderly Head and Neck Squamous Cell Carcinoma Patients Treated With Definitive Radiation Therapy. Int J Radiat Oncol Biol Phys. 2017; 98:793–801.
58. Weiss BG, Ihler F, Wolff HA, Schneider S, Canis M, Steiner W, Welz C. Transoral laser microsurgery for treatment for hypopharyngeal cancer in 211 patients. Head Neck. 2017 May 5.
59. Tam S, Araslanova R, Low TH, Warner A, Yoo J, Fung K, MacNeil SD, Palma DA, Nichols AC6. Estimating Survival After Salvage Surgery for Recurrent Oral Cavity Cancer. JAMA Otolaryngol Head Neck Surg. 2017 Apr 27.
60. Shi X, Zhang TT, Hu WP, Ji QH,2. Marital status and survival of patients with oral cavity squamous cell carcinoma: a population-based study. Oncotarget. 2017; 8:28526–28543. https://doi.org/10.18632/oncotarget.16095.
61. Arunkumar G, Murugan AK, Prasanna Srinivasa Rao H, Subbiah S, Rajaraman R, Munirajan AK. Long non-coding RNA CCAT1 is overexpressed in oral squamous cell carcinomas and predicts poor prognosis. Biomed Rep. 2017; 6:455–462.
62. Choi ES, Oh S, Jang B, Yu HJ, Shin JA, Cho NP, Yang IH, Won DH, Kwon HJ, Hong SD, Cho SD4. Silymarin and its active component silibinin act as novel therapeutic alternatives for salivary gland cancer by targeting the ERK1/2-Bim signaling cascade. Cell Oncol (Dordr). 2017; 40:235–246.
63. Kim JD, Chang JT, Moghaddamjou A, Kornelsen EA, Ruan JY, Olson RA, Cheung WY2. Asian and non-Asian disparities in outcomes of non-nasopharyngeal head and neck cancer. Laryngoscope. 2017 Apr 11.
64. Iqbal MS, Chaw C, Kovarik J, Aslam S, Jackson A, Kelly J, Dobrowsky W, Kelly C. Primary Concurrent Chemoradiation in Head and Neck Cancers with Weekly Cisplatin Chemotherapy: Analysis of Compliance, Toxicity and Survival. Int Arch Otorhinolaryngol. 2017; 21:171–177.
65. Jain V, Sekhon R, Pasricha S, Giri S, Modi KB, Shrestha E, Ram D, Rawal S. Clinicopathological Characteristics and Prognostic Factors of Synchronous Endometrial and Ovarian Cancers-A Single-Institute Review of 43 Cases. Int J Gynecol Cancer. 2017; 27:938–946.
66. Oronsky B, Ray CM, Spira AI, Trepel JB, Carter CA, Cottrill HM6. A brief review of the management of platinum-resistant-platinum-refractory ovarian cancer. Med Oncol. 2017; 34:103.
67. May T, Comeau R, Sun P, Kotsopoulos J, Narod SA, Rosen B, Ghatage P. A Comparison of Survival Outcomes in Advanced Serous Ovarian Cancer Patients Treated With Primary Debulking Surgery Versus Neoadjuvant Chemotherapy. Int J Gynecol Cancer. 2017; 27:668–674.
68. Zhang XY, Zhang PY2. Recent perspectives of epithelial ovarian carcinoma. Oncol Lett. 2016; 12:3055–3058.
69. Huang Y, Zhang W, Wang Y. The feasibility of fertility-sparing surgery in treating advanced-stage borderline ovarian tumors: A meta-analysis. Taiwan J Obstet Gynecol. 2016; 55:319–25.
70. Zhou J, Shan G, Chen Y3. Jpn J Clin Oncol. 2016; 46:718–26.
71. Van Le L, McCormack M. Enhancing Care of the Survivor of Gynecologic Cancer: Managing the Menopause and Radiation Toxicity. Am Soc Clin Oncol Educ Book. 2016; 35:e270–5.
72. Coukos G, Tanyi J, Kandalaft LE3. Opportunities in immunotherapy of ovarian cancer. Ann Oncol. 2016;27 Suppl 1:i11-i15.
73. Langhe R. microRNA and Ovarian Cancer. Adv Exp Med Biol. 2015; 889:119–51.
74. Samuel P, Pink RC, Brooks SA, Carter DR. miRNAs and ovarian cancer: a miRiad of mechanisms to induce cisplatin drug resistance. Expert Rev Anticancer Ther. 2016; 16:57–70.
75. Duchesne GM, Woo HH, Bassett JK, Bowe SJ, D'Este C, Frydenberg M, King M, Ledwich L, Loblaw A, Malone S, Millar J, Milne R, Smith RG, et al. Timing of androgen-deprivation therapy in patients with prostate cancer with a rising PSA (TROG 03.06 and VCOG PR 01-03 [TOAD]): a randomised, multicentre, non-blinded, phase 3 trial. Lancet Oncol. 2016; 17:727–37.
76. Rueda-Camino JA, Losada-Vila B, De Ancos-Aracil CL, Rodríguez-Lajusticia L, Tardío JC, Zapatero-Gaviria A. Small cell carcinoma of the prostate presenting with

Cushing Syndrome. A narrative review of an uncommon condition. Ann Med. 2016; 48:293–9.

77. Krpina K, Markić D, Rahelić D, Ahel J, Rubinić N, Španjol J. 10-year survival of a patient with metastatic prostate cancer: Case report and literature review. Arch Ital Urol Androl. 2015; 87:252–3.

78. Li B, Thrasher JB, Terranova P3. Glycogen synthase kinase-3: a potential preventive target for prostate cancer management. Urol Oncol. 2015; 33:456–63.

79. Chen C, Lin T, Zhou Y, Li D, Xu K, Li Z, Fan X, Zhong G, He W, Chen X, He X, Huang J. Adjuvant and salvage radiotherapy after prostatectomy: a systematic review and meta-analysis. PLoS One. 2014; 9:e104918.

80. Yashi M, Mizuno T, Yuki H, Masuda A, Kambara T, Betsunoh H, Abe H, Fukabori Y, Muraishi O, Suzuki K, Nakazato Y, Kamai T. Prostate volume and biopsy tumor length are significant predictors for classical and redefined insignificant cancer on prostatectomy specimens in Japanese men with favorable pathologic features on biopsy. BMC Urol. 2014; 14:43.

81. Deshmukh RR, Schmitt SM, Hwang C, Dou QP. Chemotherapeutic inhibitors in the treatment of prostate cancer. Expert Opin Pharmacother. 2014; 15:11–22.

82. McNeill SA, Good DW, Stewart GD, Stolzenburg JU. Five-year oncological outcomes of endoscopic extraperitoneal radical prostatectomy (EERPE) for prostate cancer: results from a medium-volume UK centre. BJU Int. 2014; 113:449–57.
